# CASE STUDIES OF A SENOR-ENHANCED CARE COORDINATION MODEL TO ADDRESS OLDER ADULT HEALTH

**DOI:** 10.1093/geroni/igad104.0315

**Published:** 2023-12-21

**Authors:** Erin Robinson, Elizabeth Heaton Curtis, Suzette Mizutani Bacon, Ashley Roberts, Elizabeth Conrow, Lori Popejoy, Amy Vogelsmeier

**Affiliations:** University of Missouri School of Social Work, Columbia, Missouri, United States; Sinclair School of Nursing, University of Missouri, Columbia, Missouri, United States; Sinclair School of Nursing, University of Missouri, Columbia, Missouri, United States; Sinclair School of Nursing, University of Missouri, Columbia, Missouri, United States; Sinclair School of Nursing, University of Missouri, Columbia, Missouri, United States; University of Missouri, Columbia, Missouri, United States; University of Missouri, Columbia, Missouri, United States

## Abstract

Smart technologies hold enormous potential in the early detection of activity change that may indicate illness or functional decline among older adults. However, little research currently exists on the application of such technologies to community-dwelling older adults. Our program, Age-friendly Sustainable Smart and Equitable Technologies for Aging in Place (ASSETs for AIP) uses open-source smart home technologies and consumer-grade wearable devices to monitor community-dwelling older adults recently discharged from the Show-Me Home program. We employ a novel approach; pairing technology with a care coordination team, comprised of a registered nurse, occupational therapist, and a licensed social worker. This presentation will highlight the function of this team, and how they leverage sensor-generated health information to provide care coordination. Using a clinical interface that displays the sensor-generated health data, this team provides ongoing monitoring of daily activity to identify early changes in routine that may indicate the development of a health problem. As significant changes in health data present, our clinical team works with clients to understand the change and formulate a plan of action. For example, an unexplained spike in heart rate may lead the clinician to discuss recent activity, exertion, and potential modifications in routine for a client. An increase in middle-of-the night bathroom trips may indicate a health change, for the which the client will be coached in what to communicate to their provider. Case studies from our program will be discussed in this presentation, to highlight this model of sensor-enhanced care coordination.

